# Choroidal measurements in patients affected by PXE-related retinopathy

**DOI:** 10.3389/fopht.2025.1647390

**Published:** 2025-10-06

**Authors:** Dario Pasquale Mucciolo, Vittoria Murro, Dario Giorgio, Federica Boraldi, Laura Pavese, Daniela Quaglino, Andrea Sodi, Marco Branchetti, Liliana Pollazzi, Gianni Virgili, Fabrizio Giansanti

**Affiliations:** 1 Department of Neuroscience, Psychology, Drug Research and Child Health, University of Florence, Florence, Italy; 2 SOC Oculistica Firenze, Azienda USL Toscana Centro, Firenze, Italy; 3 Department of Life Sciences, University of Modena and Reggio Emilia, Modena, Italy; 4 Struttura Complessa di Malattie Oftalmologiche, Azienda Ospedaliero Universitaria di Modena, Modena, Italy; 5 IRCCS-Fondazione Bietti, Rome, Italy

**Keywords:** pseudoxanthoma elasticum, PXE, choroidal vascularity index, OCT, choroid, *ABCC6*

## Abstract

**Purpose:**

To investigate the choroid in patients affected by pseudoxanthoma elasticum (PXE)-related retinopathy using the choroidal vascularity index (CVI).

**Methods:**

PXE patients and controls were recruited at the Eye Clinic in Florence. High-resolution imaging optical coherence tomography (OCT) scans (12 × 9 mm) of 32 PXE patients and 20 age-matched controls were examined. Images were binarized using the ImageJ software, and subfoveal choroidal thickness (SFCT), luminal area (LA), stromal area (SA), total choroidal area (TCA), and CVI were measured.

**Results:**

Sixty-four eyes of 32 PXE patients (mean age 45.65 ± 16.12; range 14–69) and 40 eyes of 20 controls (mean age 47.3 ± 13.7; range 18–71) were included in the study. SFCT was significantly lower in PXE patients compared to controls. The LA, SA, and TCA of the PXE patients were significantly reduced in comparison with those obtained for controls (p = 0.012, p < 0.001, and p = 0.001, respectively). On the contrary, the CVI did not significantly differ between patients and controls (p = 0.744). In young subjects, differences regarding SFCT, LA, SA, TCA, and CVI were not detected between PXE patients and healthy controls (p = 0.170, p = 0.990, p = 0.264, p = 0.351, and p = 0.487, respectively).

**Conclusion:**

In PXE-related retinopathy, choroidal impairment appears progressive with age, and there is a simultaneous, proportional impairment of both the stromal and vascular components of the choroid.

## Introduction

Pseudoxanthoma elasticum (PXE; OMIM# 264800) is a rare disease with an estimated prevalence of 1:25,000, typically caused by biallelic pathogenic variants on the *ABCC6* gene (1–4). Typical fundus abnormalities in patients affected by PXE are angioid streaks, peau d’orange/coquille d’oeuf, comet lesions, and optic nerve head drusen, whereas late-onset features are pattern dystrophy-like changes (5), chorioretinal atrophy, and choroidal neovascularization (CNV) (6–8). Although its exact pathophysiology is unknown, the disease is characterized by systemic calcification and fragmentation of elastic fibers in several organs (9), which also affects Bruch’s membrane (BM) (10, 11). Although the BM is thought to be the most affected eye structure in PXE patients (12–14), previous reports have suggested that the choroid is also involved in the pathogenesis of the disease (15, 16), even if a detailed characterization of the changes occurring in this tissue is lacking. With the advent of optical coherence tomography (OCT) technology, such as enhanced depth imaging (EDI) or swept-source OCT, the visualization of the choroidal structure along with a more accurate measurement of several quantitative parameters is now available. Among these, the measurement of the choroidal thickness has recently gained increasing interest. In fact, few works have analyzed the variations of the choroidal thickness in patients affected by PXE, and they report conflicting results (17, 18). However, this parameter appears to be mostly influenced by different factors such as axial length and aging, revealing that the measurement of the choroidal thickness seems not to be a sensitive biomarker to study choroidal changes.

The choroidal vascularity index (CVI), defined as the ratio of the luminal area (LA) to the total choroidal area (TCA), is a novel parameter that could be used as an alternative biomarker to monitor choroidal changes (19, 20). More precisely, the CVI has been widely used to image the choroid in several diseases such as pachychoroid neovasculopathy, central serous chorioretinopathy, panuveitis, age-related macular degeneration, and choroideremia (19, 21–23).

The analysis of the CVI may give us more detailed information about the whole choroidal structure and provide additional insights into choroidal changes by quantifying both luminal and stromal choroidal components.

The aim of our work was to evaluate the CVI and other OCT-based choroidal parameters in patients affected by PXE and compare them with those in healthy controls.

## Materials and methods

In this retrospective cohort study, patients were recruited from the Regional Reference Center for Hereditary Retinal Degenerations at the Careggi Teaching Hospital in Florence. Thirty-two patients affected by PXE were recruited and compared to 20 age- and ethnicity-matched healthy controls.

Subjects were age-matched using the last available examination (if there was follow-up). The latest time-point was also used to produce graphs of age vs. OCT variables.

The present study was conducted in agreement with the Declaration of Helsinki and was reviewed and approved by an institutional review board (local ethics committee, “Comitato Etico Regionale per la sperimentazione clinica della Regione Toscana”) (Project ID: 2018/13014) before the study began. Written informed consent was obtained from each patient before enrollment in both study groups.

The diagnostic criteria for PXE included the presence of characteristic skin alterations, ocular fundus features, genetic test examination, and/or histopathologic findings in dermal biopsies. The genetic examination was performed at the Department of Life Sciences, University of Modena and Reggio Emilia. Rare sequence variants in the *ABCC6* gene were detected by Sanger sequencing on genomic DNA isolated from whole blood (QIAamp blood kit, Qiagen GmbH, Hilden, Germany) as previously described (5). Skin biopsies obtained from 10 patients were examined. Samples were fixed in 2.5% glutaraldehyde (Electron Microscopy Sciences, Hatfield, PA, USA) in 0.1 M cacodylate buffer at pH 7.4 and successively post-fixed in 1% osmium tetroxide using the same buffer. After dehydration, samples were embedded in Spurr (Electron Microscopy Sciences). Ultrathin sections were mounted on 150 mesh copper grids (Electron Microscopy Sciences) and observed using a Talos F200S G2 transmission electron microscope (Thermo Fisher Scientific, Waltham, MA, USA) (24).

**Figure 1:**
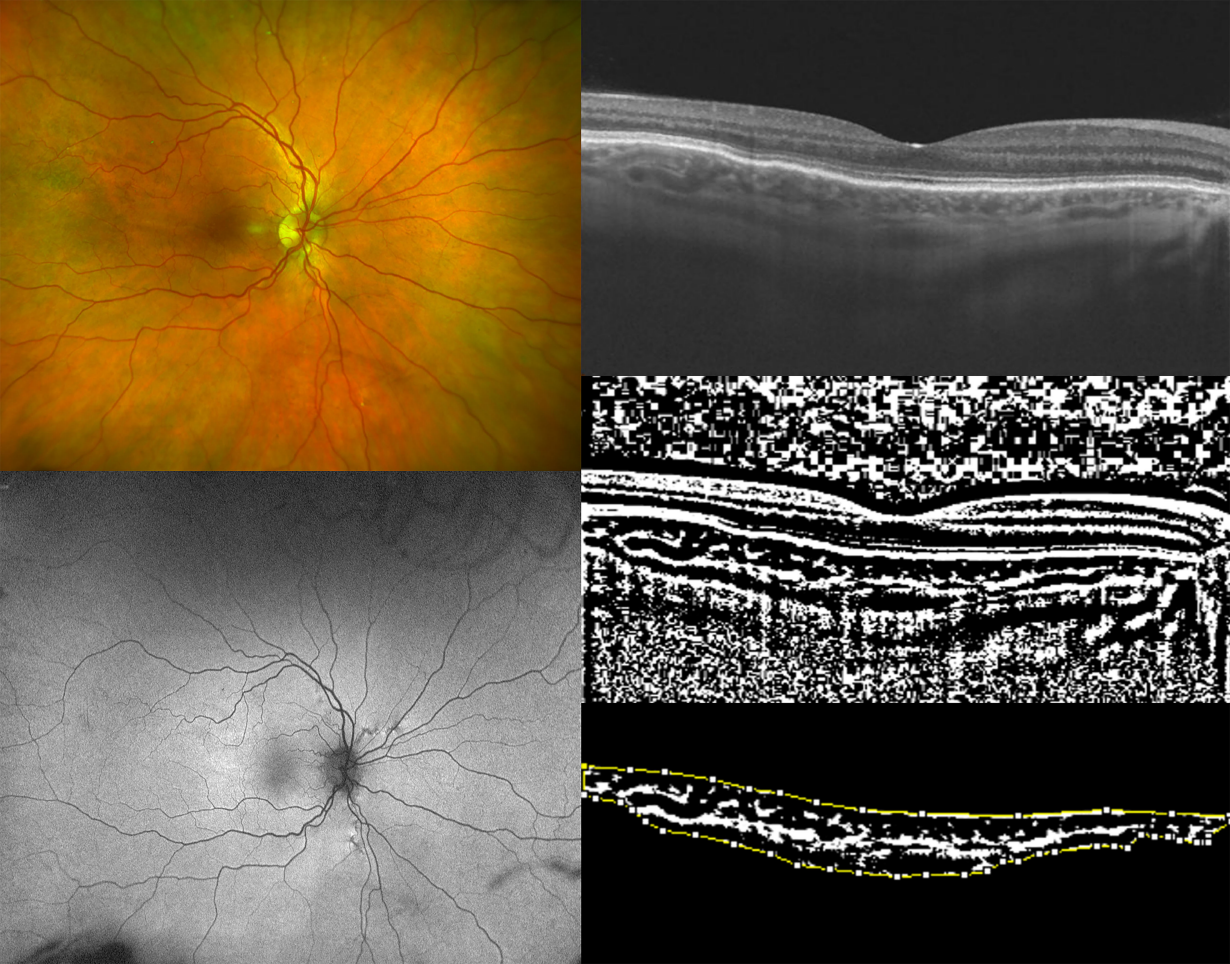
Ultra-widefield (UWF) color fundus imaging (the top-left image) and UWF fundus autofluorescence of the right eye (OD) of a 50-year-old woman affected by pseudoxanthoma elasticum (PXE) (the bottom-left image). Horizontal B-scan (12×9mm) optical coherence tomography (OCT) (the top-right image) passing through the fovea showing the appearance of a preserved external limiting membrane (ELM), ellipsoid zone (EZ), and retinal pigment epithelium (RPE) layer with undulation of Bruch’s membrane. The image was binarized using Niblack’s auto-local threshold (the middle-right image) in order to calculate the choroidal vascularity index (CVI). The dark pixels represent the luminal area and the white pixels the stromal area. The CVI was computed by dividing the luminal area (LA) by the total choroidal area (TCA). After uploading the images on ImageJ, a polygon tool was used to select the TCA (area between the yellow lines),with the RPE as the anterior boundary of TCA and the sclero-choroidal interface as the posterior boundary of TCA, across the entire length of the scan (the bottom-right image).

**Figure 2:**
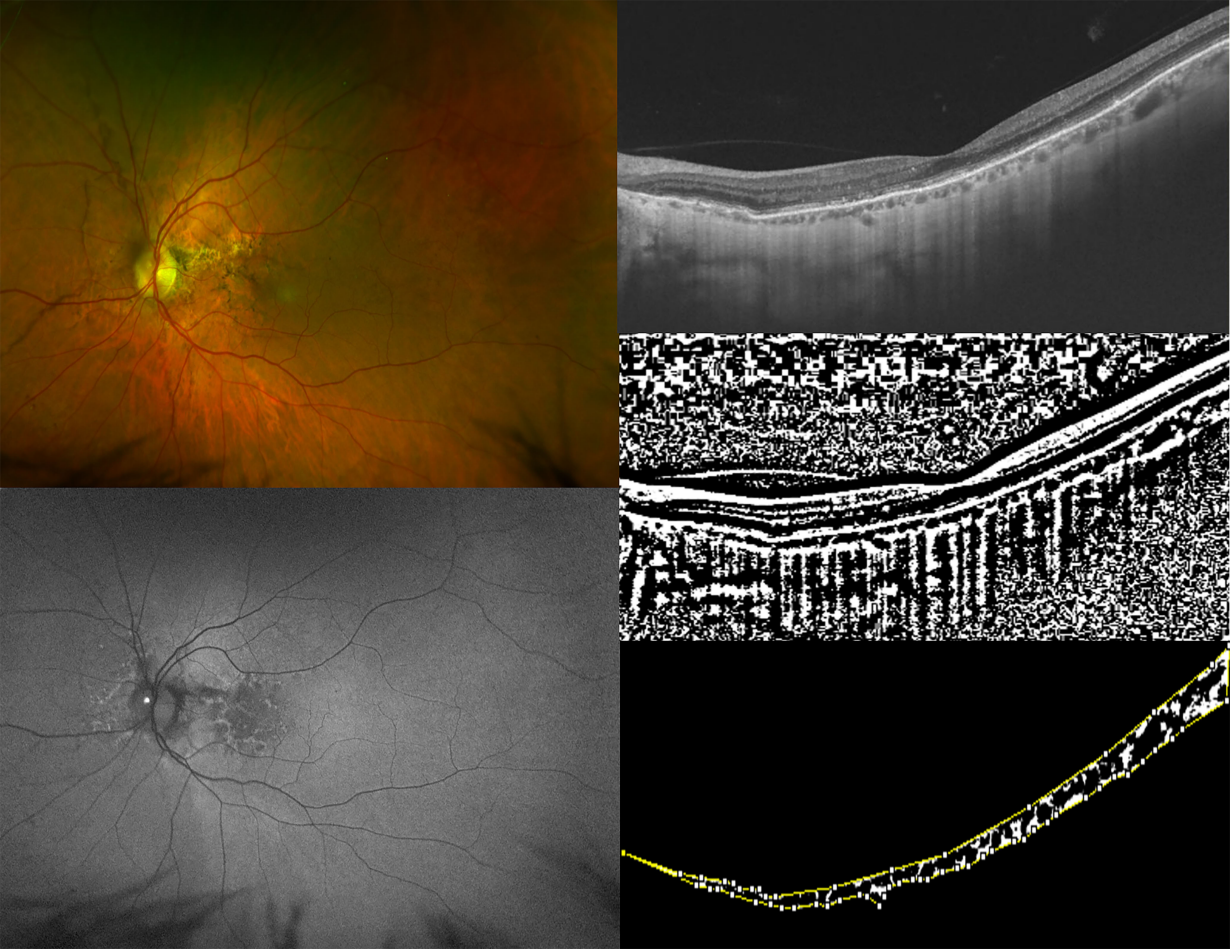
Ultra-widefield (UWF) color fundus imaging (the top-left image) and UWF fundus autofluorescence of the left eye (LE) (the bottom-left image) of a 60-year-old woman affected by pseudoxanthoma elasticum (PXE). UWF-fundus autofluorescence shows pattern dystrophy like changes and optic nerve head drusen in the LE of the patient. Horizontal B-scan (12×9mm) optical coherence tomography (OCT) (the top right image) passing through the fovea showing irregularities of external limiting membrane (ELM), ellipsoid zone (EZ), and retinal pigment epithelium (RPE) layer with Bruch’s membrane undulation. The image was binarized using Niblack’s auto-local threshold in order to calculate the choroidal vascularity index (CVI). The dark pixels represent the luminal area and the white pixels the stromal area (the middle-right image). TheCVI was calculated as the ratio of the luminal area (LA) to the total choroidal area (TCA). After uploading the images on ImageJ, a polygon tool was used to select TCA (area between the yellow lines), with the RPE as the anterior boundary of TCA and the sclero-choroidal interface as the posterior boundary of TCA, across the entire length of the scan. (the bottom-right image).

As part of the standard clinical examination, all patients and controls underwent a comprehensive ophthalmological examination, including best corrected visual acuity (BCVA), biomicroscopy of the anterior segment, and fundus examination after dilation with tropicamide 1% eye drops. Color fundus photography (FF450 Retinograph, Carl Zeiss Meditec, Jena, Germany), fundus autofluorescence (FAF), (ultra-widefield digital scanning laser technology, Daytona, Optos, Dunfermline, Scotland, UK), and OCT examinations (DRI OCT Triton; Topcon Corporation, Tokyo, Japan) were obtained. A high-resolution horizontal OCT scan across the fovea was obtained using the swept-source (SS) OCT scans (DRI-OCT Triton; Topcon Corporation, Tokyo, Japan) and chosen for the analysis. Subfoveal choroidal thickness (SFCT) was manually measured at the fovea by two independent, experienced readers (V.M and DP.M) using the caliper tool included in the device. To be precise, SFCT was measured as the distance between Bruch’s membrane [located at the lower edge of the retinal pigment epithelium (RPE)] and the sclera-choroidal interface. The OCT images were binarized and segmented by the same examiners using a public domain software “ImageJ software” (https://imagej.net/Welcome) and using a method previously described (19): the OCT images were opened in ImageJ, and the polygon tool was used to select the region of interest (ROI) across the entire length of the OCT scan. The upper boundary of the ROI was traced along the choroidal-retinal pigment epithelium junction and the lower boundary along the choroidal-scleral junction to identify the TCA. After conversion to an 8-bit image, Niblack’s auto-local threshold was applied in order to binarize the image and demarcate the LA and the stromal area (SA) (a window size/radius of 15 pixels and a k-value of 0.0 were applied); then, the image was converted using the color threshold tool in order to select dark to white pixels. The TCA and the LA were then measured (**Figures 1**, **2**).

To be precise, the TCA was defined as the total area of the ROI examined. The LA was represented by the black pixels and the SA by the white pixels. The SA was measured by subtracting the LA from the TCA. In particular, all measurements were performed independently by two experienced researchers, LP and DG, blinded to the clinical data. Measurements were repeated twice for each image, and the average was used. The CVI was then calculated as the ratio of the LA to the TCA. The mean value of these parameters was considered for the statistical analysis. In order to compare OCT variables between groups, age was centered at its gross mean value (45.6 years) in linear mixed models accounting for within-subject correlation between eyes, as well as multiple time-points, when available. We made this choice since we considered that centering at the mean of overall age improves the robustness of our prediction of age-related changes, avoiding making inference at the border of the data distribution. Values are expressed as mean ± SD. In all analyses, values p ≤ 0.05 were considered statistically significant. In the study, both patients and controls were identified using an anonymous ID. Statistical analyses were performed using Stata version 18.5 (StataCorp, College Station, TX, USA).

## Results

**Figure 3:**
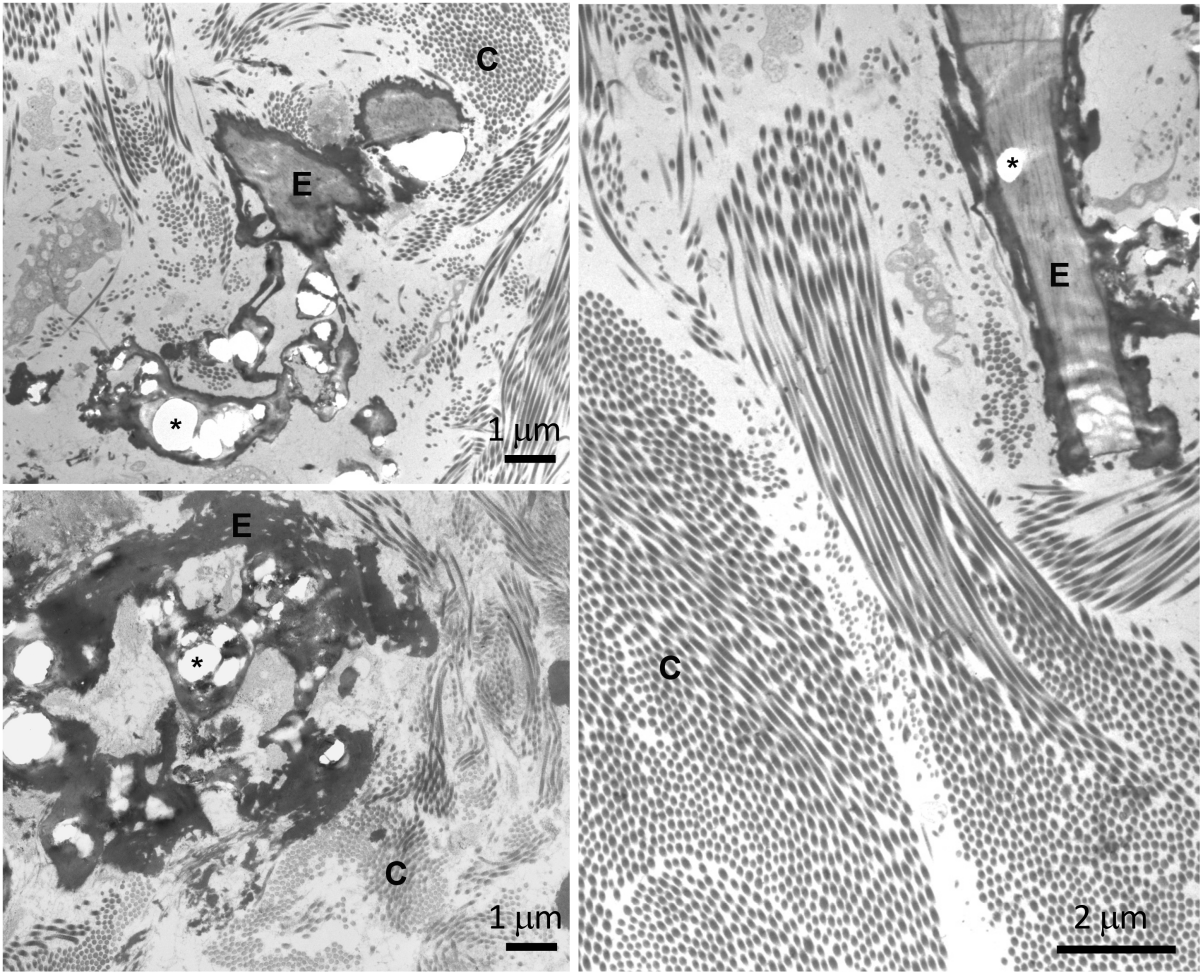
Transmission electron microscopy of skin biopsies obtained from three pseudoxanthoma elasticum (PXE) patients (top left, bottom left ad right panels) showing collagen bundles (C) and elastic fibers (E). Calcified areas (asterisk) cause evident deformities of the amorphous structure of the fibers.

All patients received a clinical diagnosis of positive PXE for at least two major phenotypic diagnostic criteria (i.e., skin and ocular manifestations) (25). To further confirm the clinical diagnosis, biomolecular tests were performed on the *ABCC6* gene. Homozygous or compound heterozygous for two rare *ABCC6* sequence variants were found in 28 patients; in three patients, the second pathogenic variant was not detected; and in one patient, DNA for molecular testing was not available. Dermal biopsy was available for 10 patients: in all cases, ultrastructural analysis revealed collagen fibrils with heterogeneous diameters and the presence of fragmented and calcified elastic fibers in the reticular dermis (**Figure 3**).

Sixty-four eyes of 32 PXE patients (mean age 45.65 ± 16.12; range 14–69) and 40 eyes of 20 controls (mean age 47.3 ± 13.7; range 18–71) were included in the study. The mean BCVA was 0.22 ± 2.56 logMAR (range 1.4–0) and 0.05 ± 0.14 logMAR (range 0.5–0) with a mean spherical equivalent (SE) of −0.66 ± 2.55 (range −8 to +7) (interquartile range −1.56 to 0) and −0.75 ± 1.89 (range −4.50 to +3) (interquartile range −1.94 to 0) for patients and controls, respectively. SE did not significantly differ between patients and controls. All eyes were phakic.

All patients presented angioid streaks (AS) and peau d’orange (PD). Comet lesions were visible in 40 eyes (40/64; 62.5%) of 20 patients, whereas six eyes (6/64; 9.3%) of four patients displayed optic nerve head drusen. Pattern dystrophy-like changes were detected in 22 eyes (22/64; 34.3%) of 11 patients, while 20 eyes (20/64; 31.2%) of 12 patients presented a history of CNV (bilateral involvement was detected in eight patients). The exact number of intravitreal injections was not available for four patients (4/12), as they had previously been followed up at other centers; for the remaining patients with at least 5-year follow-up, the average number of injections per year was 6.8 (considering both eyes).

In these patients, the type of the *ABCC6* sequence variants (e.g., nonsense or missense) was heterogeneous, and therefore, it was not possible to relate the genotype to the severity of the clinical phenotype or the presence/absence of the pattern dystrophy. The clinical and genetic findings are summarized in **Table 1**.

**Table 1:** Clinical and genetic findings of PXE patients and controls.

ID	Age	SE OD	SE OS	BCVA OD	BCVA OS	Mutation 1				Mutation 2				Reference
						Intron/exon	Nucleotide variation	Amino acid variation	ACMG pathogenicity	Intron/exon	Nucleotide variation	Amino acid variation	ACMG pathogenicity	
P1	40	1.50	1.00	0.0	0.0	Ex 12	c.1552C>T	p.Arg518Ter	Pathogenic	Ex 24	c.3421C>T	p.Arg1141Ter	Pathogenic	(26, 27)
P2	53	5.00	5.50	0.1	0.1	Ex 8	c.956T>A	p.Ile319Asn	Likely pathogenic	Ex 27	c.3774_3775insC	p.Trp1259LeufsTer19	Pathogenic	(5, 28)
P3	50	−1.00	−0.75	0.0	0.0	Ex 12	c.1553G>A	p.Arg518Gln	Pathogenic	Ex 18	c.2278C>T	p.Arg760Trp	Likely pathogenic	(29, 30)
P4	39	0.00	0.00	0.0	0.0	Ex 14	c.1857dupC	p.Ser620LeufsTer121	Pathogenic	Ex 18	c.2294G>A	p.Arg765Gln	Pathogenic	(28, 31)
P5	17	−2.00	−3.50	0.0	0.0	Ex 24	c.3490C>T	p.Arg1164Ter	Pathogenic	IVS 26	c.3736-1G>A	Loss of splice acceptor site	Pathogenic	(32, 33)
P6	15	−0.50	−0.25	0.0	0.0	Ex 24	c.3490C>T	p.Arg1164Ter	Pathogenic	IVS 26	c.3736-1G>A	Loss of splice acceptor site	Pathogenic	(32, 33)
P7	30	0.00	0.00	0.0	0.0	Ex 18	c.2263G>A	p.Gly755Arg	Pathogenic	Ex 23	c.3088C>T	p.Arg1030Ter	Pathogenic	(28, 31)
P8	66	−2.25	−2.50	0.0	1.0	Ex 24	c.3421C>T	p.Arg1141Ter	Pathogenic	Ex 29	c.4198G>A	p.Glu1400Lys	Pathogenic	(27, 34)
P9	63	0.00	0.00	0.8	0.9	Ex 18	c.2278C>T	p.Arg760Trp	Likely pathogenic	Ex 18	c.2278C>T	p.Arg760Trp	Likely pathogenic	(30)
P10	53	1	0.50	0.5	0.3	Ex 12	c.1526C>G	p.Ala509Gly	Likely pathogenic	Ex 24	c.3491G>A	p.Arg1164Gln	Likely pathogenic	(35, 36)
P11	58	0	0	0.0	0.1	Ex 24	c.3421C>T	p.Arg1141Ter	Pathogenic	Ex 24	c.3421C>T	p.Arg1141Ter	Pathogenic	(27)
P12	58	0	0	0.0	0.0	Ex 10	c.1308G>A	p.Trp436Ter	Pathogenic	Ex 10	c.1308G>A	p.Trp436Ter	Pathogenic	(37)
P13	44	0	0	0.0	0.0	Ex 24	c.3421C>T	p.Arg1141Ter	Pathogenic	Ex 29	c.4070G>C	p.Arg1357Pro	Likely pathogenic	(27, 37)
P14	64	0	0	1.1	0.6	Ex 21	c.2728_2746dupTGGATGACCCTGACAGGGC	p.Trp918Ter	Pathogenic	IVS 26	c.3736-1G>A	Loss of splice acceptor site	Pathogenic	(33, 37)
P15	60	−1	−1.25	0.1	0.0	Ex 24	c.3341G>A	p.Arg1114His	Likely pathogenic	Ex 25	c.3542G>A	p.Gly1181Asp	Likely pathogenic	(38, 39)
P16	40	0	0	0.0	0.0	Ex12	c.1553G>A	p.Arg518Gln	Pathogenic	Ex12	c.1553G>A	p.Arg518Gln	Pathogenic	(29)
P17	14	0	0	0.0	0.0	Ex 9	c.1132C>T	p.Gln378Ter	Pathogenic	not found				(40)
P18	48	0	0	0.4	0.1	Ex 14	c.1798C>T	p.Arg600Cys	Likely pathogenic	not found				(41)
P19	66	−2.75	−4.75	0.8	0.7	Ex 9	c.1132C>T	p.Gln378Ter	Pathogenic	Ex 9	c.1132C>T	p.Gln378Ter	Pathogenic	(40)
P20	48	−0.50	−0.75	0.1	0.0	Ex 24	c.3380T>C	p.Met1127Thr	Likely pathogenic	Ex 27	c.3880_3882delAAG	p.Lys1294del	Likely pathogenic	(42)
P21	39	−1.50	−1.50	0.0	0.0	Ex 12	c.1552C>T	p.Arg518Ter	Pathogenic	Ex 24	c.3421C>T	p.Arg1141Ter	Pathogenic	(26, 27)
P22	38	−2.25	−2.5	0.4	1.4	Ex 9	c.1132C>T	p.Gln378Ter	Pathogenic	Ex 9	c.1132C>T	p.Gln378Ter	Pathogenic	(40)
P23	48	0	0	0.0	0.0	Ex 26	c.3661C>T	p.Arg1221Cys	Likely pathogenic	IVS 26	c.3736-1G>A	Loss of splice acceptor site	Pathogenic	(33, 43)
P24	58	−2.5	−0.75	0.0	0.0	Ex 29	c.4198G>A	p.Glu1400Lys	Pathogenic	not found				(34)
P25	51	−4.5	−3	0.0	0.0	Ex 24	c.3490C>T	p.Arg1164Ter	Pathogenic	Ex 23_29	c.2996_4208del	p.?	Pathogenic	(31, 32)
P26	65	0.75	0.75	0.8	0.0	no DNA								
P27	44	−1.5	−1.75	0.0	0.0	Ex 12	c.1553G>A	p.Arg518Gln		Ex 12	c.1553G>A	p.Arg518Gln	Pathogenic	(29)
P28	22	−8	−8	0.1	0.1	IVS 17	c.2247 + 1G>A	Loss of splice donor site	Pathogenic	Ex 26	c.3661 C>T	p.Arg1221Cys	Likely pathogenic	(43, 44)
P29	22	−5	−4.50	0.0	0.0	IVS 17	c.2247 + 1G>A	Loss of splice donor site	Pathogenic	Ex 26	c.3661 C>T	p.Arg1221Cys	Likely pathogenic	(43, 44)
P30	25	0	0	0.0	0.0	Ex 24	c.3413G>A	p.Arg1138Gln	Likely pathogenic	Ex 24	c.3413G>A	p.Arg1138Gln	Likely pathogenic	(33)
P31	54	7	6	0.0	0.1	Ex 8	c.951C>A	p.Ser317Arg	Pathogenic	Ex 27	c.3871delG	p.Ala1291GlnfsTer68	Pathogenic	(37, 45)
P32	69	−1	0.5	1.1	1.3	Ex 24	c.3421C>T	p.Arg1141Ter	Pathogenic	Ex 24	c.3421C>T	p.Arg1141Ter	Pathogenic	(27)
C1	46	1	0.50	0.0	0.0									
C2	51	−0.5	−0.75	0.0	0.0									
C3	43	0	0	0.0	0.0									
C4	53	−2.50	−2.50	0.0	0.0									
C5	52	−0.25	0	0.0	0.0									
C6	18	0	0	0.0	0.0									
C7	52	−2.75	−3	0.0	0.0									
C8	60	1	1	0.5	0.4									
C9	55	1.50	1.25	0.0	0.0									
C10	54	−1.00	−1.50	0.0	0.0									
C11	55	2.50	3.00	0.4	0.5									
C12	60	0.00	0.00	0.0	0.0									
C13	53	−1.25	−1.75	0.0	0.0									
C14	71	0.00	0.00	0.0	0.0									
C15	56	0.00	0.00	0.0	0.0									
C16	28	0.00	0.50	0.0	0.0									
C17	27	−4.00	−4.00	0.0	0.0									
C18	29	−4.00	−4.00	0.0	0.0									
C19	30	−4.50	−4.25	0.0	0.0									
C20	53	0.00	0.00	0.0	0.0									

PXE, pseudoxanthoma elasticum; BCVA, best corrected visual acuity; SE, spherical equivalent; ACMG, American College of Medical Genetics and Genomics.

Regarding mean values of OCT parameters, SFCT was significantly lower in PXE patients compared to controls (218.47 ± 91.17 µm vs. 277.05 ± 59.63 µm; p = 0.031). The LA, SA, and TCA of PXE patients were significantly reduced in comparison to those obtained in controls (0.846 ± 0.29 vs. 1.056 ± 0.21 mm^2^, p = 0.012; 0.382 ± 0.08 vs. 0.510 ± 0.11 mm^2^, p < 0.001; and 1.230 ± 0.36 vs. 1.567 ± 0.31 mm^2^, p = 0.001, respectively). On the contrary, the CVI did not significantly differ between patients and controls (67.60 ± 5.20 vs. 67.58 ± 3.69; p = 0.744) (**Table 2**).

**Table 2:** SFCT, LA, SA, TCA, and CVI between patients and controls.

	PXE patients	Controls	P-value
Age (years)	45.65 ± 16.12	47.3 ± 15.29	0.635
SE (diopters)	−0.66 ± 2.56	−0.76 ± 1.89	0.845
SFCT (µm)	218.47 ± 91.17	277.05 ± 59.63	**0.031**
LA (mm^2^)	0.846 ± 0.29	1.056 ± 0.21	**0.012**
SA (mm^2^)	0.382 ± 0.08	0.510 ± 0.11	**<0.001**
TCA (mm^2^)	1.230 ± 0.36	1.567 ± 0.31	**0.001**
CVI	67.60 ± 5.20	67.58 ± 3.69	0.744

Significant values (<0.05) are in bold.

SE, spherical equivalent; SFCT, subfoveal choroidal thickness; LA, luminal area; SA, stromal area; TCA, total choroidal area; CVI, choroidal vascularity index.

Furthermore, we took into consideration young patients (≤30 years of age), and we did not detect differences in the values of the SE, SFCT, LA, SA, TCA, and CVI between PXE patients and healthy controls (**Table 3**).

**Table 3:** SFCT, LA, SA, TCA, and CVI between PXE patients and controls younger than 30 years.

	PXE patients (≤30 years) (n = 7)	Controls (≤30 years) (n = 5)	P-value
Age (years)	20.7 ± 5.7	26.4 ± 4.8	0.102
SE (diopters)	−2.27 ± 2.56	−2.24 ± 2.20	0.890
SFCT (µm)	299.07 ± 74.1	258.4 ± 61.51	0.170
LA (mm^2^)	1.043 ± 0.31	1.044 ± 0.19	0.990
SA (mm^2^)	0.431 ± 0.07	0.471 ± 0.34	0.264
TCA (mm^2^)	1.475 ± 0.38	1.516 ± 0.27	0.351
CVI	69.89 ± 3.63	68.96 ± 2.39	0.487

SE, spherical equivalent; SFCT, subfoveal choroidal thickness; LA, luminal area; SA, stromal area; TCA, total choroidal area; CVI, choroidal vascularity index; PXE, pseudoxanthoma elasticum.

**Table 4** shows the differences in OCT parameters between PXE patients and controls at the sample mean age. As explained in the Materials and Methods, they were obtained from a model investigating whether each OCT parameter regression slope differed with age between PXE patients and controls; this was carried out by fitting a group-by-age interaction term.

**Table 4:** Predicted values of the choroidal parameters at the mean age (45.6 years) and linear changes per 10 years of age of SFCT, LA, SA, TCA, and CVI between the study groups.

Variable	Predicted values at mean age 45.6 years		Linear change per 10 years of age		
	Control	PXE (difference, 95% CI)	Control	PXE	Interaction with age β coefficient (95% CI) p-Value
SFCT (µm)	276.4	−50.6 (−91.1 to −10)*	7.1 (−16.7 to 30.9)	−28.8* (−43.4 to −14.2)	−35.9 (−63.8 to −7.9) 0.012*
LA (mm^2^)	1.06	−0.19 (−0.33 to −0.05)	0 (−0.01 to 0.01)	0.01 (−0.07 to 0.10)	−0.09 (−0.19 to 0.00) 0.059
TCA (mm^2^)	1.57	−0.31 (−0.49 to −0.13)**	0 (−0.1 to 0.01)	−0.09** (−0.16 to −0.02)	−1.1 (−2.3 to 0.01) 0.083
CVI	67.6	3.66 (−19.2 to 26.5)	0.05 (−13.0 to 13.9)	−1.31 (−2.16 to −0.46)**	−1.4 (−3.0 to 0.24) 0.095
SA (mm^2^)	0.51	−0.12 (−0.18 to −0.07)**	0 (−0.03 to 0.04)	−0.14 (−0.03 to 0.05)	−0.02 (−0.5 to 0.02) 0.297

SFCT, subfoveal choroidal thickness; LA, luminal area; SA, stromal area; TCA, total choroidal area; CVI, choroidal vascularity index; PXE, pseudoxanthoma elasticum.

(*) p < 0.05; (**) p < 0.01.

**Figure 4:**
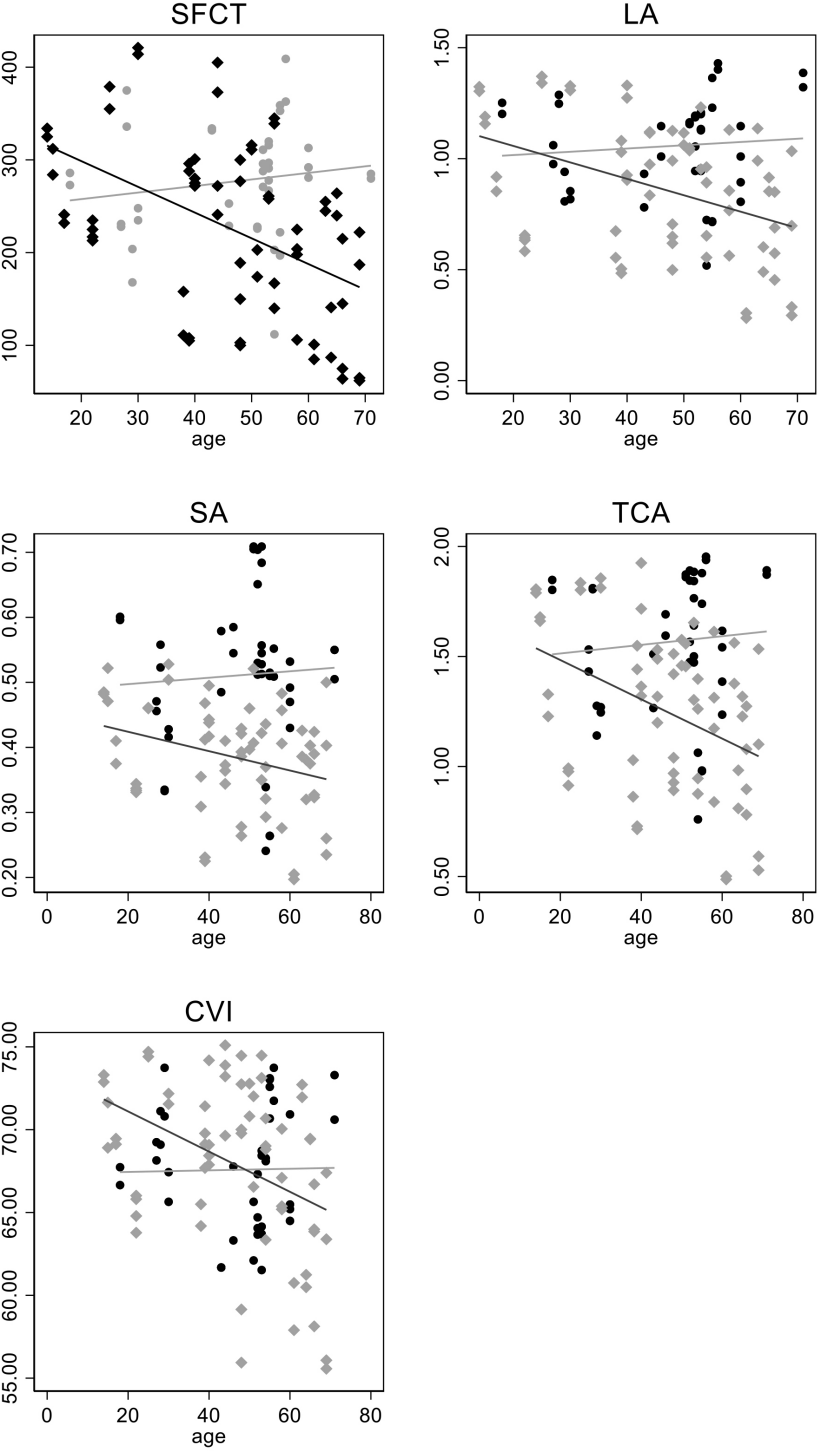
The scatterplot and linear fit of the relationship between optical coherence tomography (OCT) parameters and age at the last follow-up.

SFCT differed by approximately 50 μm at the mean age of 46.5 years (p = 0.015). Moreover, the group difference in SFCT decreased with age by −29 μm per 10 years (p = 0.012 for group * age interaction). Significantly lower values of the TCA and SA were also recorded between groups at the mean sample age (p < 0.01). In addition, no OCT parameter showed a significant decrease with age, whereas this was detected for SFCT and also for the TCA and CVI in PXE patients. The consistency of the findings was remarkable for all OCT parameters despite the fact that the group-by-age interaction term was significant only for SFCT and of borderline significance (0.1 < p < 0.05) for the LA, TCA, and CVI; **Figure 4** shows the scatterplot and linear fit of the relationship between OCT parameters and age at the last follow-up.

## Discussion

In this study, we used the novel binarized technique applied to high-definition OCT scans to investigate the changes occurring in the choroid in PXE patients and to compare them with those in healthy controls. To the best of our knowledge, this is the first study that provides an extensive analysis of the choroidal tissue using the CVI in PXE patients.

In agreement with previous studies (16, 17, 46, 47), we found that SFCT was significantly lower in PXE patients compared to controls. In addition, in our work, we observed that the LA, SA, and TCA were significantly reduced compared to those in controls; therefore, the entire choroidal tissue (vascular and stromal components) was reduced in PXE patients, suggesting that choroidal angiopathy represents a process of PXE-related retinopathy. The exact mechanism of this choroidal thinning is not completely understood, even if changes in the diseased Bruch’s membrane are considered the primary pathologic alteration. More specifically, Gliem et al. (17) revealed that choroidal thinning seemed to follow the centrifugal pattern of BM calcification, suggesting that BM calcification can affect choroidal thickness and is responsible for atrophy of the outer retina and the RPE (5, 48). BM calcification probably impedes the diffusion of factors secreted by the RPE, such as vascular endothelial growth factor (VEGF), which are essential for maintaining the integrity of the choroid (17, 47); in fact, histopathologic works have reported atrophic changes and disruptions of the choroid in areas of angioid streaks (49, 50) and suggested a characteristic loss of the choriocapillaris underneath a thickened and calcified BM, while choroidal vessels showed no specific alterations (51). In particular, our study revealed that the choroidal vessels of medium and large caliper were also involved due to the LA and SA abnormalities in PXE patients compared to controls.

From our results, the CVI did not differ between PXE patients and controls. Therefore, we observed a substantial proportional reduction of the vascular network of the choroid and stromal tissue in PXE patients compared to healthy subjects (CVI represents the ratio of the LA to the TCA). This result confirms the hypothesis that Bruch’s membrane could be responsible for the choroid abnormalities: as the vascular and stromal components of the choroid were reduced in the same proportion, it is plausible that this phenomenon could be attributable to the absence of trophic factors derived from the RPE (due to calcified Bruch’s membrane).

In our study, we focused on a subgroup analysis, taking into consideration younger patients (≤30 years of age): all OCT-based choroidal parameters (SFCT, LA, SA, TCA, and CVI) did not differ between the PXE patients and controls; however, this finding must be interpreted with caution due to the limited statistical power resulting from the small sample size in the young patient subgroup. These results suggested that when Bruch’s membrane already shows signs of calcification (as happens in young PXE patients) (14), the choroidal tissue is not yet involved, and normal choroidal vasculature and stroma suggested that BM calcification precedes choroidal changes. These results confirm the pathogenetic hypothesis previously explained about the primary role of a calcified Bruch’s membrane.

In order to compare OCT variables between groups, we centered age at its gross mean value (45.6 years) in linear mixed models accounting for within-subject correlation between eyes as well as multiple time-points, when available.

SFCT showed a significant decrease with age; furthermore, this was also detected for the TCA and LA in PXE patients [significant only for SFCT and of borderline significance (0.1 < p < 0.05) for the TCA and LA]. These data are very interesting because they tell us that the choroidal tissue showed progressive damage in PXE-related retinopathy. Moreover, in elderly people affected by PXE, the CVI seems to decrease with age (it was not statistically significant); this result could be due to the difficult evaluation and measurement of choroidal thickness (and of the LA, SA, and TCA) when the choroidal thickness becomes very thin, as happens in elderly PXE patients. Longitudinal studies are required to confirm all these results. Our study has several strengths, including the use of a standardized data collection, the large sample size for a rare inherited retinal disease such as PXE, and the inclusion of an age- and gender-matched control group.

An important limitation of the study is that the calculation of choroidal parameters was based on single B-scan images rather than volumetric choroidal data; other studies have calculated the CVI using volumetric data derived from multiple consecutive scans or 3D reconstruction (52). This volumetric method may provide a more comprehensive assessment of the entire choroidal structure, potentially capturing regional variations that could be missed in single-scan evaluations. However, it also requires more complex image processing and longer acquisition times, which may not always be feasible in clinical settings. Moreover, volumetric approaches are more dependent on the overall image quality: motion artifacts, poor fixation, or media opacities may affect multiple scans and compromise the reliability of the data. In addition, volumetric protocols are less standardized compared to single subfoveal B-scans, as they vary in terms of grid size, number of scans, and whether segmentation is manual or automated, making inter-study comparisons more difficult. Another limitation is the absence of axial length data, which were not routinely collected. Since choroidal thickness is known to be influenced by AL, the lack of this parameter may represent a potential confounding factor. Furthermore, due to the axial resolution limitations of OCT, subtle differences in choroidal vascularity may not be captured, potentially contributing to the lack of statistically significant differences in the CVI.

In conclusion, our work permitted us to make important considerations: in PXE-related retinopathy, there is a simultaneous, proportional impairment of both the stromal and vascular components of the choroid, and choroidal impairment appears progressive with age.

## Data Availability

The datasets presented in this study can be found in online repositories. The names of the repository/repositories and accession number(s) can be found in the article/supplementary material.
